# Rare Presentation of Pseudotumor Cerebri in a Pediatric Patient With Granulomatosis With Polyangiitis

**DOI:** 10.7759/cureus.11567

**Published:** 2020-11-19

**Authors:** Henry J Sioufi, Shayan Makvandi, Rana Masoud, Xiaoyan Wu, Rabheh Abdul-Aziz

**Affiliations:** 1 Pediatrics, Oishei Children's Hospital/University at Buffalo, Buffalo, USA; 2 Pediatric Nephrology, Jacobs School of Medicine and Biomedical Sciences, University at Buffalo, Buffalo, USA; 3 Pediatric Rheumatology, Jacobs School of Medicine and Biomedical Sciences, University at Buffalo, Buffalo, USA

**Keywords:** pseudotumor cerebri, pediatric gpa, granulomatosis with polyangiitis, wegner’s granulomatosis, idiopathic intracranial hypertension (iih)

## Abstract

We present a case of an 11-year-old female with granulomatosis with polyangiitis (GPA) diagnosed one year prior to presentation, stage III chronic kidney disease (CKD), hypertension (HTN), obstructive sleep apnea (OSA), and chronic anemia who presented with headache, nausea, vomiting, and diplopia and was found to have idiopathic intracranial hypertension (IIH). IIH was reported in adults with GPA and for our knowledge has not been yet reported in pediatric cases of GPA.

## Introduction

Granulomatosis with polyangiitis (GPA), formerly known as Wegner’s granulomatosis, is a rare autoimmune vasculitis affecting small and medium arteries, arterioles, and capillaries [1]. In GPA anti-neutrophil cytoplasmic antibodies (ANCA) form against a neutrophilic enzyme called proteinase-3 which causes unregulated inflammation. This leads to the development of granulomatous necrotizing vasculitis, particularly targeting the blood vessels of the lungs and kidneys [2-3]. While GPA most commonly affects middle-aged adults, the symptoms can manifest at any age [4]. Treatment is targeted at halting the inflammatory processes of the dysregulated immune system. The course of GPA has been dramatically improved by recent treatment, however, disease and treatment-related morbidity is often profound [4]. Pseudo-tumor cerebri, also known as idiopathic intracranial hypertension (IIH), is associated with papilledema, headaches, vision changes, and tinnitus in the setting of normal cerebrospinal fluid (CSF) and imaging studies [5]. The etiology of IIH is secondary to either increased CSF production or an impairment of CSF absorption [6]. The excess accumulation of CSF and increase in the intracranial pressure (ICP) can be debilitating and lead to multiple pathologies [7]. IIH was reported in adults with GPA and for our knowledge has not been yet reported in pediatric cases of GPA. We are describing a case of pediatric GPA complicated with IIH.

## Case presentation

We present the case of an 11-year-old female with GPA diagnosed one year prior to presentation, stage III chronic kidney disease (CKD), hypertension (HTN), obstructive sleep apnea (OSA), and chronic anemia who presented with headache, nausea, vomiting, and diplopia and was found to have IIH.

**Figure 1:**
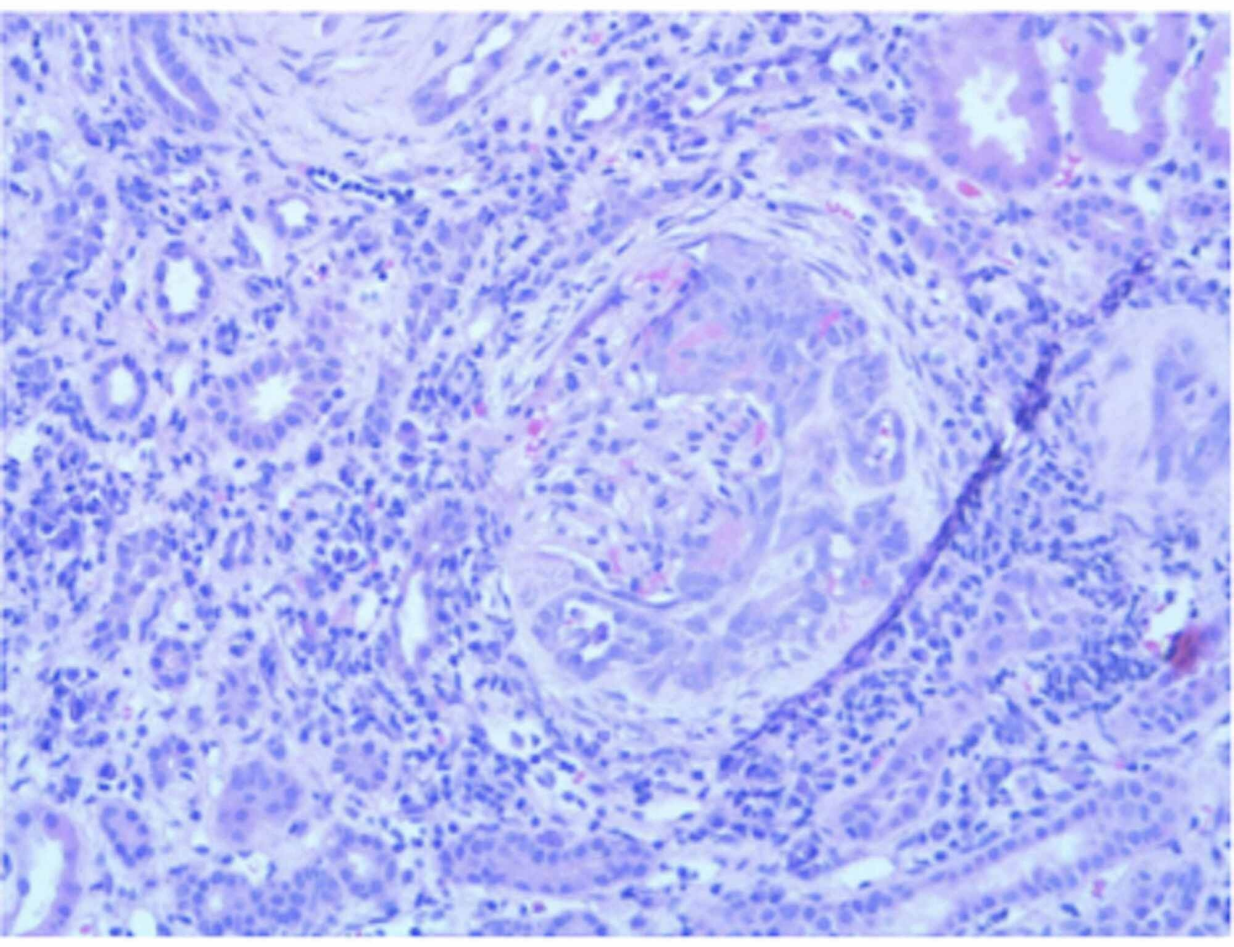
Pauci-immune glomerulonephritis.

The patient initially presented at the age of 10 with intermittent abdominal pain, nausea, and vomiting for about one year. She had a previous history of sleep apnea and nose bleeding. Exam showed paleness, HTN, ankles arthritis, rashes, and saddle nose. Work up showed anemia with hemoglobin 8.8 g/dL (normal 12-16 g/dL), elevated erythrocyte sedimentation rate (ESR) 127 mm/hr (normal 0-20 mm/hr), elevated C-reactive protein (CRP) 103 mg/L (normal <10 mg/L), elevated potassium, elevated creatinine 3.26 mg/dL (normal 0.6-1.2 mg/dL), estimated glomerular filtration rate (eGFR) 18 mL/min/1.73 m^2, elevated blood urea nitrogen (BUN) 45 mg/dL (normal 7-20 mg/dL), urinalysis with +3 hemoglobin and +3 protein, protein to creatinine ratio 2.88 (normal <0.2), positive antinuclear antibody (ANA) 1/160, positive antineutrophil cytoplasmic antibodies (ANCA) 1/640, negative myeloperoxidase (MPO), and positive PR-3 at 188 U/mL. Kidney biopsy showed pauci-immune glomerulonephritis (Figure 1). Chest CT was prominent for minimal patchy ground glass opacity and two equivocal nodular densities in the lower lobes. Sinus CT scan was prominent for nasal septum perforation. Mild mucosal thickening was noticeable in the left maxillary antrum. She had a normal echocardiogram. She was diagnosed with GPA based on the American College of Rheumatology criteria and treated successfully with IV methylprednisolone, oral steroid taper over one year, seven-month induction treatment regimen with cyclophosphamide, and started later on rituximab. She was also on enalapril for HTN and iron for anemia. She became symptom free and blood work showed normalizing of her ESR, CRP, BUN, and PR-3. ANCA became negative. She continued to have elevated urine protein/creatinine ratio 0.6 and serum creatinine around 0.9 mg/dL with eGFR 50-60 mL/min/1.73 m^2 which places her in stage III CKD.

**Figure 2:**
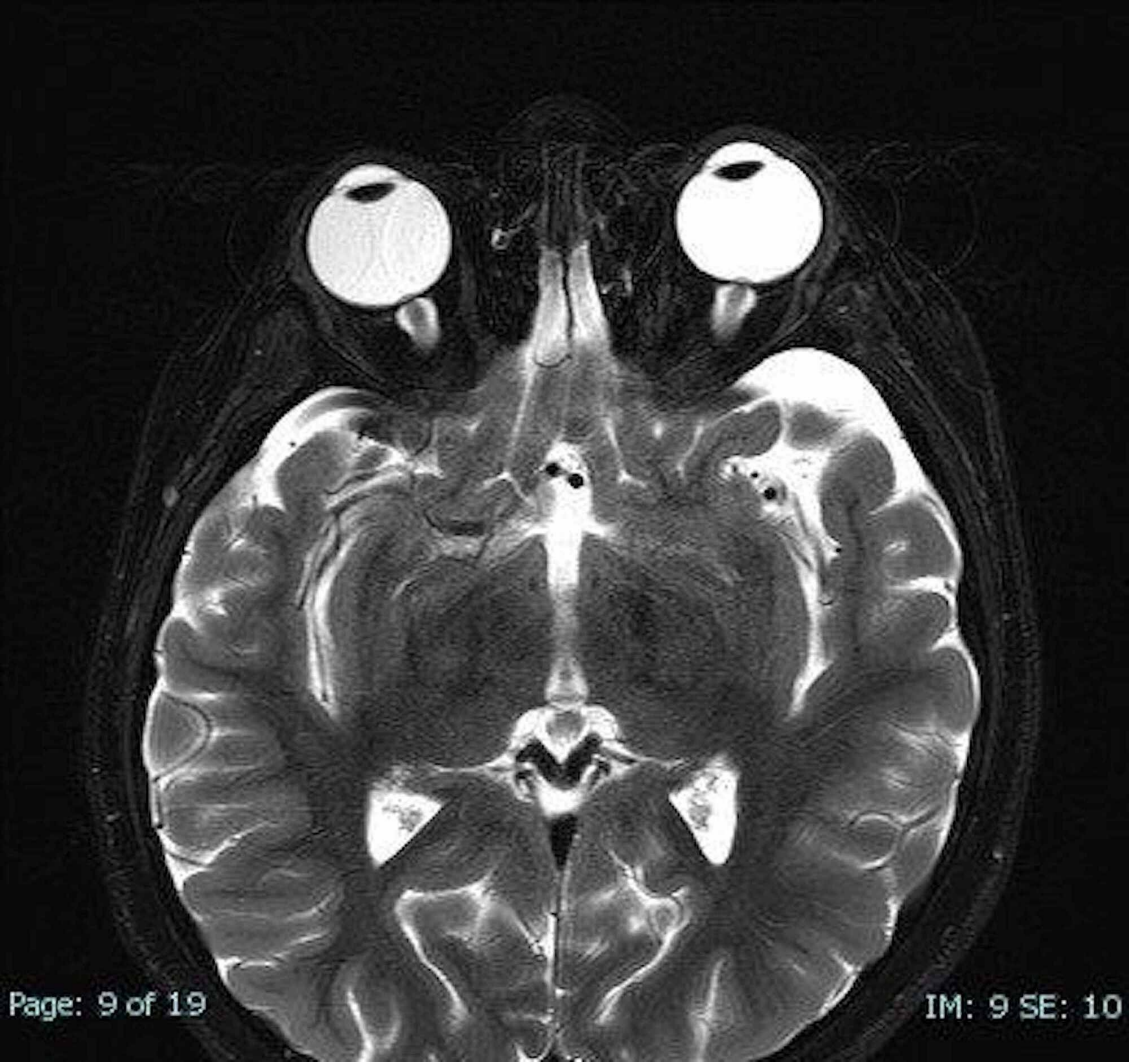
Orbital MRI exhibiting bulging of the optic nerves.

**Figure 3:**
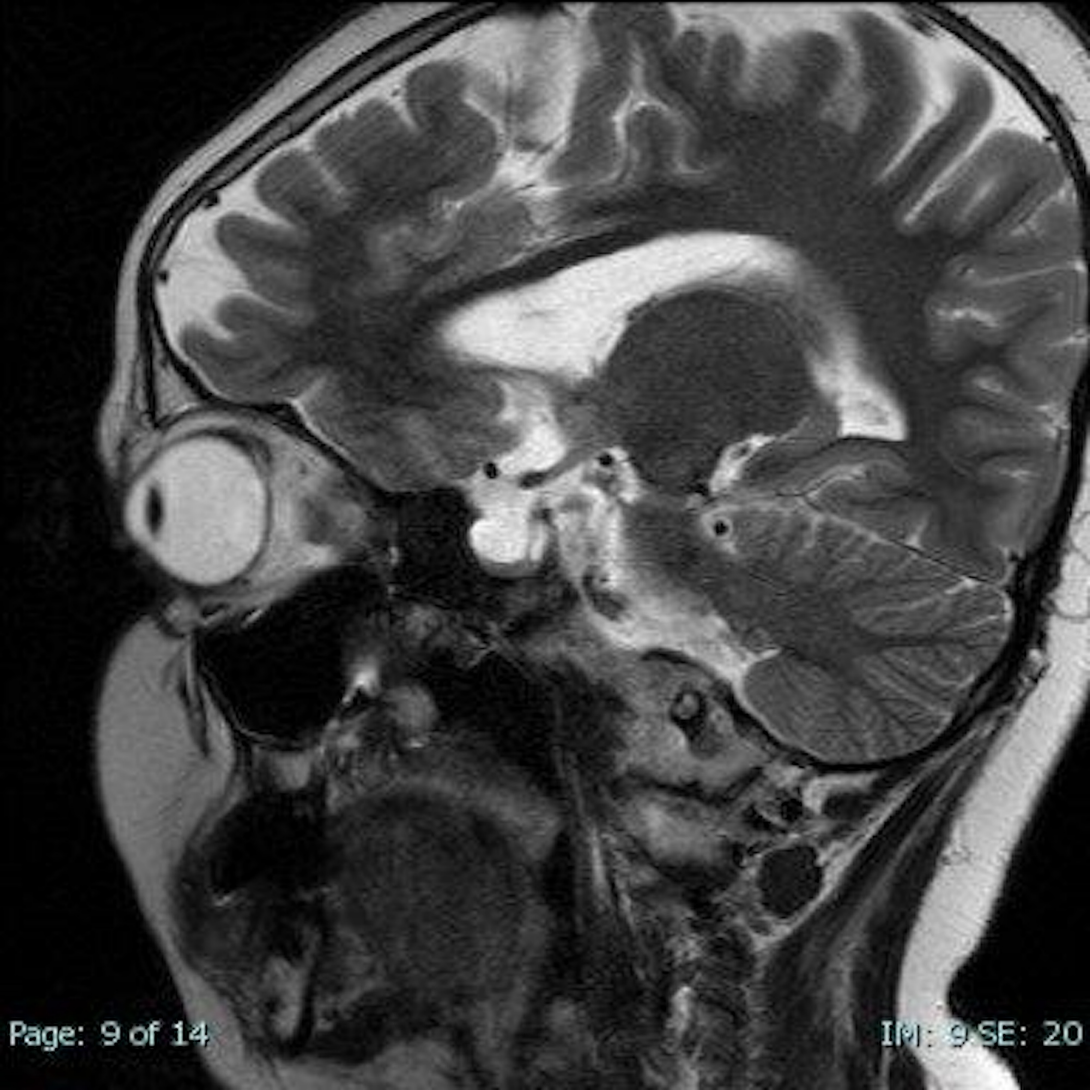
+/- Sagittal view exhibiting empty sella.

A year later since the diagnosis, she presented with vomiting and headaches. Completed work up did not show any GPA flare up. Brain MRI with and without contrast was unremarkable. A couple of days later, she developed double vision and left cranial nerve VI palsy. Exam showed bilateral papilledema. MRI orbit revealed tortuosity of the optic nerves with optic nerves bulging, apparent transverse sinus stenosis, and empty sella (Figures 2-3). Lumbar puncture was prominent for elevated opening pressure at 36 cm H2O (normal 7-18 cmH2O). CSF analysis was not consistent with any infectious etiology with normal nucleated cells, glucose, and protein. Magnetic resonance venography (MRV) showed focal stenosis of the lateral right transverse sinus and diffuse narrowing of the left transverse sinus. Subsequently, the patient was diagnosed with IIH and was started on acetazolamide 5 mg/kg twice daily which eventually was titrated to 10 mg/kg for persistence of papilledema and headaches. The patient was maintained on acetazolamide throughout the admission with gradual improvement of symptoms. Upon discharge, the patient was back at baseline without double vision or vomiting and improvement of headaches.

## Discussion

Granulomatosis with polyangiitis is a systemic necrotizing vasculitis affecting small and medium sized blood vessels [3]. The most common clinical manifestations of childhood GPA at onset are related to upper airway involvement (82%), nephropathy (65%), and lower respiratory tract disease (61%) [8]. Central nervous system (CNS) involvement is found in about 10%-45% of cases. CNS involvement was predominantly dominated by headaches, sensory or motor impairments, vestibular syndrome, and hearing loss [9]. This is the first documented case of a young preteen girl with a previous diagnosis of GPA who presented with nausea, vomiting, headache, and diplopia. This raises concerns for broad differential in a previously diagnosed GPA patient. Differentials such as, but not limited to, meningitis, GPA flare up, and pseudo-tumor cerebri. Clinical suspicion for meningitis occurs when a patient presents with sudden onset fever, headache, vomiting, and meningismus [10]. An early diagnosis and management of meningitis is critical in GPA patients to prevent detrimental outcomes of the disease. An early lumbar puncture is indicated which in our case was remarkable only for an increased opening pressure with normal cell analysis, ruling out an infectious etiology.

The GPA flare ups can occur suddenly with initial warning signs of involvement in the sinuses, throat, or lungs [2]. Our patient who complained of a severe headache raises concerns of a possible GPA flare up with pachymeningitis. In addition, her initial presentation at the time of diagnosis of GPA was significant of abdominal pain and vomiting. She presented with vomiting one year after diagnosis raised concern for GPA flare up. A complete work up ruled out GPA flare up. She had normal kidney function, ESR, CRP, PR3, and negative ANCA. Pseudo-tumor cerebri (IIH) can present with headache, vision changes, and papilledema with a completely normal CSF analysis [7]. While it has been reported that adults with GPA have had concomitant IIH, no cases have been reported in the pediatric population. It is not clear in this case if IIH has an association with GPA or due to other risk factors like recent wean of steroids and having OSA. However, it is important to keep IIH in differential diagnosis for a patient with GPA presenting with CNS involvement as treatment will be different and will affect the outcome in a patient with critical diagnosis of GPA.

## Conclusions

The unique aspect of this case is the finding of IIH in a pediatric patient with GPA. To our knowledge, these findings have only been documented in the adult population, and not in the pediatric population. It is very important to rule out other pathologies that may present in a similar fashion to IIH to minimize the risk of adverse events. The treatment method we used was acetozolamide that was initially given at 5 mg/kg and titrated up to 10 mg/kg. Our patient achieved full remission of her IIH. 
